# Investigations on the Occurrence and Associated Risk Factors of Avian Coccidiosis in Osun State, Southwestern Nigeria

**DOI:** 10.1155/2017/9264191

**Published:** 2017-08-24

**Authors:** Shola David Ola-Fadunsin

**Affiliations:** Department of Veterinary Pathology and Microbiology, Faculty of Veterinary Medicine, Universiti Putra Malaysia (UPM), 43400 Serdang, Selangor, Malaysia

## Abstract

Avian coccidiosis is one of the most important diseases of poultry and it is responsible for a large number of poultry mortalities worldwide. This study was carried out to investigate the occurrence and associated risk factors of avian coccidiosis in Osun State, Nigeria. Fecal samples were collected and examined from 5,544 avian species that were brought for treatment at the state veterinary hospitals over a 10-year period. Parameters such as age, sex, season, and species of birds were determined. Also, the months of the year were taken into consideration. Overall prevalence of 41.3% was recorded. The year specific rate for avian coccidiosis was highest in 2007 (97.9%) and lowest in 2006 (0.4%), while the month-specific rate was highest in November (85.7%) and lowest in July 2006 (13.3%). There was a significantly (*P* < 0.05) higher prevalence in young birds compared to adults, in males compared to females, and during the wet season compared to the dry season. Broilers (99.8%) and cockerels (81.0%) were the bird types with the highest prevalence rate. The high prevalence of avian coccidiosis in the study area shows that the disease is endemic and there is need to embark on a radical preventive measure to curtail the disease.

## 1. Introduction

Avian species refers basically to domestic birds such as chickens (including indigenous chickens, broilers, layers, and cockerels), turkeys, ducks, guinea fowls, peasants, pigeons, and more recently ostriches that are kept for meat or egg production [1, 2]. Poultry production contributes significantly to the socioeconomic development of many developing countries of the world [3]. In Nigeria, it is an important component of the livestock subsector and has developed to the level of commercial enterprise involving thousands of birds which provides employment, income, and animal protein for urban and rural dwellers as well as manure for crop production [4]. It is an important instrument for alleviating problems associated with poverty in Nigeria and other developing countries (in terms of food security and malnutrition). Also, it significantly contributes to household's income and helps meet some level of its protein needs [5]. Nigeria has the largest poultry population in Africa with an estimated poultry population of about 130–150 million chickens [3].

Avian coccidiosis is one of the most important diseases of poultry worldwide [6]. It is ubiquitous where chickens are reared (traditional, industrial, or organic/biofarms). It is recognized as the parasitic disease that has the greatest economic impact on poultry industries throughout the world [7]. Avian coccidiosis is an important enteric parasitic disease of poultry associated with significant economic losses to poultry farmers [8]. The parasites multiply in the intestine resulting in tissue damage, lowered feed intake, poor absorption of nutrients from the feed, dehydration, blood loss, and death [6, 9]. The global cost of the protozoan to the poultry industry is estimated to be over US$2.4 billion per annum [7, 10]. These costs involve medications to the chickens, losses due to mortality, morbidity, and poor growth of the chickens surviving the disease [7]. About 1,800* Eimeria *species have been reported to colonize and infect the intestinal tract of different animals and birds [8, 11] and infection with this pathogen normally occurs through ingestion of feed or water contaminated with sporulated oocysts [8, 12]. In domesticated birds, at least nine species of* Eimeria *have been recognized [13].* Eimeria tenella *and* E. necatrix *are the most pathogenic species;* Eimeria acervulina*,* E. maxima, *and* E. mivati *are common and moderately pathogenic;* E. brunetti *is uncommon but pathogenic when it occurs, while* E. mitis, E. praecox, *and* E. hagani *are relatively nonpathogenic species [14, 15]. Coccidiosis is usually characterized with diverse kinds of clinical signs such as dysentery, enteritis, diarrhea (which may be bloody with certain* Eimeria *species), emaciation, lower feed conversion rate, delayed sexual maturity, drooping wings, poor growth, low production, and even death [16, 17].

To date there seems to be no report on the prevalence of avian coccidiosis in Osun State, southwestern Nigeria. However, studies on the prevalence of avian coccidiosis have been conducted in other parts of Nigeria by researchers such as Grema et al. [18], Davou et al. [19], and Lawal et al. [8] who carried out their studies in Gombe, Ilorin, and Maiduguri, respectively. This present investigation was therefore undertaken to provide current information on the occurrence of avian coccidiosis in Osun State, utilizing ten-year (2006–2015) data from veterinary hospitals to determine the distribution and risk factors associated with its occurrence in the study area.

## 2. Materials and Method

The study was conducted at the zonal veterinary hospitals located in Osogbo, Ilesa, Ikirun, and Ede, Osun State, southwest Nigeria, between January 2006 and December 2015. Osun State covers an area of 9,026 square kilometers and is located between latitude 7° 30′N and longitude 4° 30′E with an altitude of 246 meters above sea level. The state is characterized by a tropical wet (March–November) and dry (December–February and August “August break from rains”) climate with a lowland tropical rain forest vegetation. The state has a mean annual rainfall of between 127.77 cm and 159.76 cm and an average annual temperature ranging from 21.1°C to 31.9°C. It records a mean relative humidity of between 58.7% and 79.6% [20].

Fecal samples collected from 5,544 avian species (3,420 chickens; 2,088 turkeys; 24 ostriches; and 12 ducks) brought for treatment at the zonal veterinary hospitals were subjected to microscopic examination (×10 objective magnification) by direct wet mount smear method for the presence of* Eimeria *oocysts as described by Soulsby [14]. Fecal samples from birds with* Eimeria *oocysts were considered positive for coccidiosis. Parameters such as age, sex, and avian species were determined. Also, the year and the month the birds were presented to the hospital were taken into consideration. The microscopic examinations and records about each bird were carried out by qualified veterinarians and laboratory scientist.

The descriptive statistics was conducted using percentages and tabulations. The univariate analysis (chi-square) test and odds ratios with 95% confidence interval were used to determine the association between each epidemiological factor and the occurrence of coccidiosis. The odds ratios were calculated with respect to a reference category as indicated in the respective tables. All statistical tests were conducted using statistical package for social sciences (SPSS) version 22 (SPSS Inc., Chicago). Significant level was set at *P* < 0.05.

## 3. Results

Out of the 5,544 avian fecal samples examined in the 10-year period, 2292 were positive for coccidiosis representing 41.3% of the population. The yearly distribution of avian coccidiosis in Osun State during the ten-year period is presented in Table 1. The number of avian coccidiosis cases was highest in 2014 (1,224 cases) and lowest in 2006 (1 case) representing 53.4% and 0.04%, respectively, of the total cases of coccidiosis. The year specific rate for avian coccidiosis was highest in 2007 (97.9%) and lowest in 2006 (0.4%). However, the odds ratios (ORs) for all the years were significant (*P* < 0.05) at 95% confidence interval in relation to the reference year 2015 (1.00) with exception of those of 2009 (0.92) and 2012 (0.82). Generally, there was no defined pattern in the total number of cases presented during the years of study.

The number of coccidiosis cases recorded was highest in October, 456 (19.9%), and December, 360 (15.7%), with the lowest one in July, 48 (2.1%), and August, 60 (2.6%). The highest month-specific rates of 85.7% and 79.2% were observed in November 72 (3.1%) and October 456 (19.9%), respectively, and lowest ones in July (13.3%) and August (16.1%) having number of coccidiosis cases of 48 (2.1%) and 60 (2.6%), respectively. The ORs of coccidiosis cases recorded in January to September were significant (*P* < 0.05) at 95% CI and lower compared to those of December (reference month), while those of October and November were not. The total number of cases peaked in February and fluctuated greatly within the other months. Seasonally, more birds were diagnosed for coccidiosis during the wet season (70.2%) compared to the dry season (29.8%) (Table 2).

The risk factors associated with avian coccidiosis in Osun State are shown in Table 3. Of the 3,552 young (not sexually mature) birds examined, 1872 were positive representing a prevalence of 52.7%, while 420 adult (sexually matured) birds were positive representing 21.1% of the total 1,992. The infection rate of coccidiosis was 4.17 times more in young birds compared to adult birds. Based on sex, male birds were 2.21 times more likely to be infected with coccidiosis compared to female birds. Of the 2,820 male birds examined 1428 (49.4%) were positive, while 864 representing 31.7% of the total 2,724 female birds were infected with coccidia protozoan. More cases were presented during the wet season compared to the dry season. Of the 3,492 cases presented in the wet season, 1,608 (46.0%) were positive for coccidiosis. Of the 2,052 cases recorded in the dry season, 684 were positive representing a prevalence of (33.3%). The infection rate of coccidiosis was 1.7 times more during the wet season compared to the dry season. Age, sex, and season were significantly (*P* < 0.05) associated with the occurrence of avian coccidiosis.

The species specific rate showed that chickens had the highest rate (58.2%) followed by turkeys (14.4%). Ducks and ostriches had rates of 8.3% and 4.2%, respectively. The coccidiosis cases of 1990 (86.8%), 300 (13.1%), 1 (0.04%), and 1 (0.04%) were recorded in chickens, turkeys, ducks, and ostriches, respectively. The ORs of all the species were significant (*P* < 0.05) at 95% CI compared to chickens (Table 4).

The distribution of avian coccidiosis within the chicken species is shown in Table 5. The highest specific rate was observed among broilers (99.8%), while cockerels, indigenous chickens, and layers had specific rates of 81.0%, 55.3%, and 39.0%, respectively. Coccidiosis cases of 576 (29.0%), 564 (28.3%), 539 (27.1%), and 311 (15.6%) were recorded in layers, indigenous chickens, broilers, and cockerels, respectively. The ORs among the chickens species were significant (*P* < 0.05) at 95% CI compared to indigenous chickens.

## 4. Discussion

Avian coccidiosis is the major cause of economic losses in the poultry industry in the world [10] and specifically in Nigeria [7]. Coccidiosis is the most common enteric parasitic disease of poultry and a major constraint to successful poultry farming in Nigeria and the world [8]. For these reasons, routine examination of feces of birds for coccidiosis is done in veterinary hospitals in Osun State, Nigeria. An overall prevalence of 41.3% was recorded in this study. This is higher compared with 36.9% in Ethiopia birds [21] and 18.4% in Pakistan birds [22]. Higher prevalence of 76.1% and 88.0% has been reported by Islam et al. [23] in Bangladesh. In other studies, the infection rate was reported to be 54.3% in Turkey [24], 39.6% in India [25], 78.0% in Jordan [26], 88.4% in Argentina [27], and 92.0% in Romania [28]. Within Nigeria, lower prevalence of 31.8%, 20.3%, 14.2%, and 11.4% has been reported by Lawal et al. [8], Davou et al. [19], Adamu et al. [29], and Grema et al. [18] in Maiduguri, Ilorin, Sokoto, and Gombe, respectively. Different factors such as sampling periods, sample size, study design, geographical area, and climatic conditions could have resulted in the disparity in the reported prevalence. Also, the difference in the prevalence rate could be attributed to the fact that more avian types were included in this study compared to the above-mentioned studies where restrictions were made to one or fewer avian types ranging between broilers, layers, duck, and local (indigenous) chickens. The relatively high prevalence reported in this work could be attributed to the high amount and duration of rainfall observed annually in the study area, as rainfall is associated with high relative humidity which favours the sporulation of* Eimeria *oocysts.

Also, the fact that most birds in the study area are raised in the deep litter system may have contributed to the high prevalence recorded.

The yearly distribution of avian coccidiosis showed no defined pattern in its prevalence rate, although the specific rate (92.7%) and the number of coccidiosis cases 1224 (53.4%) of 2014 are alarming and higher than other prevalence rates reported in Nigeria by Lawal et al. [8], Grema et al. [18], and Adamu et al. [29]; this may be attributed to differences in the geographical areas and climatic conditions. Interestingly, the high specific rate recorded in 2014 tallies with the prevalence rate reported by Györke et al. [28] in their work done in Romania.

The undefined yearly and monthly pattern of coccidiosis could be attributed to the inability of poultry farmers to consistently and properly maintain a standard biosecurity and hygienic practice in their farms. It may also be due to the involvement of more persons coming into poultry farming as a means of livelihood with most of them operating a backyard poultry system. Furthermore, the off and on participation of farmers in the poultry business, the previous avian influenza outbreak in Nigeria, and the increased sensitization of poultry farmers to report clinical cases of poultry diseases to veterinary hospitals and clinics could have contributed to this undefined pattern of distribution. The monthly specific rates of 13.3% and above, recorded all through the months and with peak prevalence observed during October and December, suggest that avian coccidiosis is endemic in Osun State, with high level of endemicity during the raining season.

Age is an important risk factor associated with the prevalence of avian coccidiosis as all ages of birds are susceptible to the disease [8]. In line with this, this study reports that both young and adult birds were infected with coccidiosis with a significant higher prevalence recorded in young birds. This finding supports earlier reports by Lawal et al. [8] and McDougald and Reid [30] who documented a higher prevalence rate of coccidiosis in birds younger than 18 weeks of age (not sexually mature) compared to older birds. The higher prevalence recorded in the young birds could be attributed to the immature immune system in young birds leaving them susceptible to infection even with the lower or less pathogenic strain of* Eimeria *species. It may also be due to the fact that most poultry farms in Osun State raise sexually immature birds on deep litters, while mature birds (especially layers) are kept in battery cages in cases where battery cage system is used. Birds raised in deep litter system have been reported to have a higher prevalence of coccidiosis compared to birds raised in battery cage [31] especially due to relatively higher accumulation of oocyst in the deep litter.

Male birds were reported to be more infected with coccidiosis compared to female birds. This finding may be attributed to the aggressive feeding nature of male birds making them pick up more sporulated oocyst from contaminated feed, water, or litters. In support of this report, Olanrewaju and Agbor [32] and Garbi et al. [33] recorded higher prevalence of coccidiosis among male chickens compared to female chickens in their studies conducted in Nigeria and Ethiopia, respectively.

Higher prevalence of avian coccidiosis has been attributed to period of rainfall [34] primarily because it positively influences the warm and humid environmental conditions needed for oocysts sporulation. This study did not report anything different as avian coccidiosis was more likely to occur during the wet season than the dry season. Previous studies by Awais et al. [17] and Grema et al. [18] had showed that avian coccidiosis is more prevalent during the raining season compared to the dry season.

Broiler birds had the highest coccidian infection rate (99.8%) compared with the other types of birds, although cockerel also had an alarming prevalence rate of 81.0%. Previous researchers in Nigeria had reported coccidia prevalence in broilers as 68.7% [8], cockerel as 70.0% [32], layers as 41.5% [31], and indigenous chickens as 18.8% [35]. Prevalence rate of 76.1% has been reported in ducks from Bangladesh [23], 66.0% in turkeys from USA [36], and 46.9% in ostriches from China [37]. The high prevalence rate recorded in the broilers birds may be attributed to the high stocking densities and intensive husbandry management systems practiced in broiler production in Osun State.

## 5. Conclusion

This study being the first to be done in Osun State gives an insight into the prevalence and risk factors associated with avian coccidiosis in the area. It can be concluded that avian coccidiosis is endemic in Osun State with higher prevalence recorded during the raining season. Age, sex, and species of birds are other important risk factors influencing the prevalence of the disease. Since avian coccidiosis exerts great negative influence on the poultry economy, there is need for proper preventive and control measures such as strict biosecurity, disinfection of poultry houses and utensils, vaccination, and good use of anticoccidial drugs.

## Figures and Tables

**Table 1:** Yearly distribution of avian coccidiosis in Osun State, Nigeria (2006–2015).

Years	Total cases (%)	Cases of coccidiosis (%)	Year specific rates (%)	OR	95% CI	*P* value
2006	276 (5.0)	1 (0.04)	0.4	0.01	0.003–0.030	<0.01^*∗*^
2007	48 (0.9)	47 (2.1)	97.9	76.36	14.57–1575.00	<0.01^*∗*^
2008	216 (3.9)	24 (1.1)	11.1	0.21	0.13–0.33	<0.01^*∗*^
2009	468 (8.4)	168 (7.3)	35.9	0.92	0.69–1.22	0.55
2010	696 (12.6)	396 (17.3)	56.9	2.16	1.66–2.81	<0.01^*∗*^
2011	540 (9.7)	36 (1.6)	6.7	0.12	0.08–0.17	<0.01^*∗*^
2012	360 (6.5)	120 (5.2)	33.3	0.82	0.60–1.11	0.20
2013	1272 (22.9)	144 (6.3)	11.3	0.21	0.16–0.28	<0.01^*∗*^
2014	1320 (23.8)	1224 (53.4)	92.7	20.80	15.44–28.17	<0.01^*∗*^
2015^a^	348 (6.3)	132 (5.8)	37.9	1.00		
*Total*	*5544 (100.0)*	*2292 (100.0)*	*41.3*			

^a^Reference category. ^*∗*^Significant. OR = odds ratio; CI = confidence interval.

**Table 2:** Monthly and seasonal distribution of avian coccidiosis in Osun State, Nigeria (2006–2015).

Months	Total cases (%)	Cases of coccidiosis (%)	Month/season specific rates (%)	OR	95% CI	*P* value
January	204 (3.7)	84 (3.7)	41.2	0.19	0.13–0.27	<0.01^*∗*^
February	1020 (18.4)	180 (7.9)	17.7	0.06	0.04–0.08	<0.01^*∗*^
March	444 (8.0)	192 (8.4)	43.2	0.20	0.15–0.27	<0.01^*∗*^
April	216 (3.9)	96 (4.2)	44.4	0.21	0.15–0.30	<0.01^*∗*^
May	588 (10.6)	276 (12.0)	46.9	0.24	0.18–0.31	<0.01^*∗*^
June	564 (10.2)	180 (7.9)	31.9	0.13	0.09–0.17	<0.01^*∗*^
July	360 (6.5)	48 (2.1)	13.3	0.04	0.03–0.06	<0.01^*∗*^
August	372 (6.7)	60 (2.6)	16.1	0.05	0.04–0.07	<0.01^*∗*^
September	660 (11.9)	288 (12.6)	43.6	0.21	0.16–0.27	<0.01^*∗*^
October	576 (10.4)	456 (19.9)	79.2	1.01	0.75–1.37	0.93
November	84 (1.5)	72 (3.1)	85.7	1.60	0.85–3.19	0.15
December^a^	456 (8.2)	360 (15.7)	79.0	1.00		
*Total*	*5544 (100.0)*	*2292 (100.0)*	*41.3*			
*Seasons*						
Wet	3492 (63.0)	1608 (70.2)	46.0	1.71	1.52–1.91	<0.01^*∗*^
Dry^a^	2052 (37.0)	684 (29.8)	33.3	1.00		
*Total*	*5544 (100.0)*	*2292 (100.0)*	*41.3*			

^a^Reference category. ^*∗*^Significant. OR = odds ratio; CI = confidence interval.

**Table 3:** Risk factors associated with avian coccidiosis in Osun State, Nigeria (2006–2015).

Risk factors	Total cases	Cases of coccidiosis (%)	OR	95% CI	*P* value
*Age*					
Young	3552	1872 (52.7)	4.17	3.68–4.73	<0.01^*∗*^
Adult^a^	1992	420 (21.1)	1.00		
*Sex*					
Male	2820	1428 (49.4)	2.21	1.98–2.46	<0.01^*∗*^
Female^a^	2724	864 (31.7)	1.00		
*Season*					
Wet	3492	1608 (46.0)	1.71	1.52–1.91	<0.01^*∗*^
Dry^a^	2052	684 (33.3)	1.00		

^a^Reference category. ^*∗*^Significant. OR = odds ratio; CI = confidence interval.

**Table 4:** Species distribution of avian coccidiosis in Osun State, Nigeria (2006–2015).

Species	Total cases (%)	Cases of coccidiosis (%)	Species specific rates (%)	OR	95% CI	*P* value
Ducks	12 (0.2)	1 (0.04)	8.3	0.07	0.01–0.38	<0.01^*∗*^
Turkeys	2088 (37.7)	300 (13.1)	14.4	0.12	0.10–0.14	<0.01^*∗*^
Ostriches	24 (0.4)	1 (0.04)	4.2	0.03	0.01–0.17	<0.01^*∗*^
Chickens^a^	3420 (61.7)	1990 (86.8)	58.2	1.00		
*Total*	*5544 (100.0)*	*2292 (100.0)*	*41.3*			

^a^Reference category. ^*∗*^Significant. OR = odds ratio; CI = confidence interval.

**Table 5:** Chickens distribution of avian coccidiosis in Osun State, Nigeria (2006–2015).

Chickens	Total cases (%)	Cases of coccidiosis (%)	Chickens specific rates (%)	OR	95% CI	*P* value
Layers	1476 (43.2)	576 (29.0)	39.0	0.52	0.44–0.61	<0.01^*∗*^
Cockerels	384 (11.2)	311 (15.6)	81.0	3.44	2.60–4.59	<0.01^*∗*^
Broilers	540 (15.8)	539 (27.1)	99.8	43.58	6.10–311.10	<0.01^*∗*^
Indigenous chickens^a^	1020 (29.8)	564 (28.3)	55.3	1.00		
*Total*	*3420 (100.0)*	*1990 (100.0)*	*58.2*			

^a^Reference category. ^*∗*^Significant. OR = odds ratio; CI = confidence interval.
